# On the Power, Wisdom, and Goodness of God, as Manifested in the Creation of Animals, and in Their History, Habits, and Instincts

**Published:** 1835-10-01

**Authors:** 


### ON THE POWER, WISDOM, AND GOODNESS OF GOD, AS MANIFESTED IN THE CREATION OF ANIMALS AND IN THEIR HISTORY, HABITS, AND INSTINCTS. By the Rev. *William Kirby*, M. A. &c. Rector of Barham. Two vols. 8vo, price Thirty Shillings.

[Bridgewater Treatise.]

WHEN will the system of jobbery, and the misapplication of public funds and private bequests have an end in this country! A ray of hope seems breaking on the moral horizon, like the sun struggling through dense clouds on a Winter morning. Whether the Trustees of the Earl of Bridgewater were *bound* by legal technicalities to *employ* individuals to write these treatises, we know not; but certainly a worse plan could not have been devised for furthering the object of the testator, than that which has been adopted. If the prizes had been held out for the *best* essays, then there would have been ardent competition; and, instead of one languid dissertation on each subject, there would probably have been a dozen—to the most meritorious of which, the donation should have been awarded. The rejected essays would all have been published, and then the object of the dying donor would have been accomplished to an infinitely greater extent than it ever can be at present. In that way, talent would have been called forth, exercised, and rewarded on a large scale—for those who did not bear off the prize, would still have reaped advantage from the cultivation of their powers in the construction of their essays.

Then, again, think of printing these treatises, designed for universal dissemination, in a style, and at an expense, that must limit their circulation to the narrowest compass. Between the lines of the work before us the Earl of Bridgewater might almost have driven his cab! Are scepticism and irreligion confined to a few of the upper classes? It would seem so, from the means which have been taken by the Earl's executors to prevent any but a few of the wealthy from having access to the Essays—Essays meant to illustrate the power, wisdom, and goodness of God! But, as usual, the whole business was a job—and the products have done little credit to this country. To the individual authors we attach no blame—nor do we mean to detract from their merits. They are all men of talent and acquirements; but the proper way of drawing forth these talents was not taken. Had they written in expectation of competition, they would all have written better. And if they were too proud to write *in competition*, we can tell them that, at least as good essays as these, would have emanated from twenty different sources.

The two costly volumes before us are, we imagine, among the best of the Bridgewater treatises. The subject of instinct—and the material organs employed by instinct, throughout the various tribes of animals, cannot be otherwise than interesting to every individual of the medical profession; but how very few can afford the expence and the time which are necessary for the purchase and perusal of these essays! On this account, we shall endeavour to seize upon, and condense the most prominent and important points of information in this work, and thus bring them within the reach of all classes and ranks of our brethren.

We shall not waste our own time, however, or the time of our readers, by dwelling on a lengthy and most unwise introduction, in which the learned and reverend gentleman endeavours to illustrate natural history by Scripture, and shadow out the whole material world—the universe itself, by the contents of the Jewish tabernacle!! Thus the "seven candlesticks" were a kind of planetarium, "representing the solar system"—the central lamp was the *sun*—the table and shew-bread were the earth and its productions, &c. &c. But Mr. Kirby is not content with materializing the furniture of the Holy of Holies,—he spiritualizes them also. The candlestick was the church and its ministers—"the lights of the world," &c. &c. We must leave him interpreting the various symbols of the Tabernacle. The author has justly ridiculed the absurd and atheistical hypotheses and speculations of La Place and Lamarck; but he himself has scarcely been less extravagant and imprudent in his attempts to explain the Jewish symbols, and square all science with the expressions in the Bible. We do not think that the cause of religion is assisted by such forced analogies and strained interpretations, but the contrary. The wisdom and goodness of the Creator are better shewn and *proved*, by a direct application to his works, where demonstration insures conviction, than by those futile attempts to make the Word of God so many oracles of science and natural knowledge, as well as of revelation.* We are truly glad, then, to take our leave of this most tedious and injudicious "introduction," which is equally calculated to make the vulgar stare and the learned grieve! This censure may seem severe. We shall, therefore, give one short extract, as a sample—and rather a favourable one—of Mr. Kirby's introduction—and if it does not prove the justice of our strictures, we acknowledge ourselves to be the very emblems of stupidity.

"The footstool of the Deity, the pavement on which his throne is placed, is over or above the heads of the cherubim; and though we cannot comprehend exactly the precise meaning of the figures employed, yet the general idea seems to be that of *irradiation;* and by these representations the claim of Jehovah the God of Israel is indicated to supremacy and entire dominion over the physical cherubim, or the heavens in a state of action, and as the sole fountain and centre of that incessant radiation and glory, and of those constant effluxes by which the whole universe of systems and worlds is maintained." xcvi.

### Chapter the First.

This is on the Creation of Animals. In a rather eloquent proemium, the author informs us that *man* was created after the world had been peopled (if we may use the expression) by the various tribes of animals—these last being preceded by vegetables. The *mode* of creation is very conveniently

---

* Professor Sedgwick has, we think, pursued a wiser plan in his "discourse on the studies of the Cambridge University." He has leant more on the proofs of wisdom and goodness in the Deity, by reference to his visible works than to revelations of inspired men.

taken from the Bible. On the *fifth day*, "the boundless ocean became prolific, and brought forth, by myriads, its destined offspring." On the same day, "the winged and feathered tribes" sprang into existence. Again, "the word of power was spoken—and instantly the various tribes of quadrupeds issued from her teeming womb." The whole animal creation is despatched in a page or two—and then, "the earth was now completely *furnished and decorated* to receive her destined king and master."

"The sun, the moon, and the stars were shedding their kindly influences upon her; she and her fellow planets had commenced their annual and diurnal revolutions; the plants and flowers, her first born progeny, had sprung out of her bosom, and covered her with verdure and beauty; and the fruit and forest trees flourishing in all their glory of leaf, blossom, and fruit, were ready to minister to the support, comfort, and enjoyment of their future lord: the sea, the air, the earth, were each filled with their appropriate inhabitants, and throughout the whole creation was beauty, and grace, and life, and motion, and joy, and jubilee." 6.

But MAN was wanting to add to this joy and jubilee! How far he contributed to increase them, let the heart, or rather the head of man conscientiously determine! The vast superiority of MAN over animals is, as usual, proved by Scripture.

"When they (animals) were brought into existence, the word was—'*Let the waters bring forth—Let the earth bring forth*,' from which it should seem that God did not act *immediately* in their creation, except by his agency on those powers that he had established as rulers in nature, and by which he ordinarily taketh hold, as it were, of the material universe. But when a being, combining the spiritual with the material world, is to be created, all the persons of the Godhead unite *immediately* in the work, and without the intervention of any other agent, '*Let* us *make man*.'" 8.

We are informed by our author that at this auspicious æra, "the generations of the world were perfect and healthful." The instincts of animals urged them to fulfil their several functions—but yet this instinct was restrained, otherwise the *carnivorous* animals would have annihilated the *herbivorous*. "They, (the tiger, lion, &c.) therefore, must have originally eaten grass or straw, like the ox." ! ! !

"And to this vegetable diet, before the close of the present scene, we are assured they shall again return so as to render the last age of the world as happy as the original state of man in Paradise." 10.

This harmony of the animal creation, our author thinks, "continued long enough, after the fall, to allow sufficient time for such a multiplication of the flocks and herds as would secure them from extinction." The author does not inform us at what precise period God altered the *organization* of the carnivorous animals, and adapted them to the digestion of vegetable food. No. He merely gives us his IPSE DIXIT that God "restrained their instincts," and ordered the incisor teeth of the tiger to grind corn and rice, till such time as sufficient numbers of kids, lambs, and other animals were multiplied to supply the altered gusto of the leopard, the lion, and the hyena!!

Has the Reverend Author never reflected on the inferences which might be drawn from such doctrines as those which he has here promulgated? If animals can thus be changed in their nature, so far that the tiger may browse on the flowery lawn with the kid, why need we shudder at the idea of the monkey taking a start of growth, at some remote period, and shooting up into man? It is a transition not a whit less feasible than the metamorphosis of a carnivorous stomach into a graminivorous! See then the folly of making physiology bend to the *literal* text of the Bible! Mr. Kirby has just as good grounds for maintaining that, in the time of Joshua, the sun revolved round the earth, as that the ferocious carnivori fed on corn at any period of the world. It is lamentable to perceive writers of Mr. Kirby's class insisting on the *literal* meaning of a word or passage in one portion of Scripture—but twisting it into a *metaphor* in the very next page of the sacred volume, and when it happens to suit their hypothesis! Cuvier and other naturalists have abundantly proved that the species of animals will never alter throughout any number of ages, but remain the same, in structure and functions for ever. They may mix for a time, and a hybrid race may be produced, but these last cannot procreate. We gladly pass over nearly one hundred and fifty pages of letter-press (independent of the introduction) in order to get at something like plain and rational information. Whenever Mr. Kirby trusts to *himself*, he wanders among the clouds, lost and bewildered in the mazes of conjecture, ever and anon stringing together long quotations from the Bible. It is when he is forced to draw his science from others, and when he permits himself to make use of his own *acquired* information on physical subjects, that he is both instructive and entertaining.

We have now had our worst SAY; but in censuring the *judgment* of our author, we are far from impugning his *motives*. We verily believe them to be of the most benevolent kind—and, without the slightest personal knowledge of Mr. Kirby, we have set him down in our own minds, as a man of single-heart, intense philanthropy, and very considerable attainments. This testimony may well console him for those criticisms (which he will no doubt consider as false) that have been directed against certain metaphysical and theological speculations in the volume before us.

From the chapters on the geographical distribution, and also the migrations of animals, we cannot condense any information suitable to our pages. It is in the fourth chapter that Mr. Kirby begins to approach his subject in earnest, in the lowest grade of animated nature—the INFUSORIA—the protozoa and protophyta. These half vegetable half animal beings, distinguished by their simple structure and oscillatory movements, resemble, when collected in masses, a piece of green velvet, covering moist surfaces, or swimming on water. The filaments are continually oscillating from right to left, or from left to right—or in opposite directions, while some of them remain stationary. Agardh, Unger, and others, think that these oscillating plants owe their existence to animalcules which, at first, swim about as animals, and afterwards fix themselves as plants. Mr. K. thinks it a contradiction to the general analogy of creation, that any creature should begin life as an animal, and end it as a plant.

Leaving these doubtful forms, however, we come to the INFUSORIA themselves—and especially the *polypes* and *zoophytes*, which our author considers as the "basis on which the Deity has built the animal kingdom." These wonderful animalcules, though every where dispersed like seeds, remain sometimes motionless, for years, till they come in contact with moisture, when they are instantly re-animated—move about in all directions, absorb nutriment, and exercise their reproductive powers. These last are very little different from those of vegetables. They are spontaneously divisible, these cuttings becoming separate animals—and they are also propagated by germs. But still, they are possessed of irritability and voluntary locomotion—consequently they are ANIMALS. Their organization proves this. They have, almost all of them, a mouth and digestive organs—many of them have eyes—and even some rudiments of a nervous system. They may be said, too, to be *omnipresent.* They are found in the sea, the rivers, the air, the blood, the urine, in animal substances, in vegetables—in every thing almost! Their numbers are infinite. Hundreds of thousands inhabit a single drop of water. They are also *proteiform.* Some of them can change their shape at pleasure.

" In nothing is the power and wisdom of their Almighty Author more signally conspicuous. *Organization so complex, and life, and spontaneous motion, and appetite, and means to satisfy it, and digestion, and nutrition, and powers of reproduction in animals of such infinite minuteness!* Who can believe it? Yet so it is, and that each of these should be varied in the different tribes and genera—that these less than the least of all the creatures that present themselves to the observation of mankind, and which till within a century or two were not suspected to exist, should out-number beyond all statement of numbers, all the other animals together that people the whole globe, that they should probably enter into us and circulate in our blood, nestle between our teeth, be busy every where, and perceived no where, till the invention of the microscope drew aside the veil between us and these entities, and we saw how God had filled all things with life, and had based the animal kingdom upon living atoms, as well as formed the earth and the world of inert ones." 158.

Our readers will perceive some degree of sublimity in the foregoing passage, mixed with some extremely defective English. Observe the second sentence, which we have marked in italics. There is a host of nominative cases, without a single verb to support them! It is really astonishing that men of such learning should be utterly inattentive to the most ordinary rules of grammar!

Our author thinks we may safely conclude that this infinite host of animalcules, " was not created merely to be born and die." We agree with him. They were created to enjoy existence, just as much as the elephant. This, to us, appears certain. The following is possible.

" With respect to its immediate action upon the vegetable and animal kingdoms, it has been ascertained, as to many species, that they ascend with the sap in vegetables, and are found in the blood and excretions of animals, who knows but they may act an important part in the animal frame; somewhat similar to what devolves upon the larves of certain insects, with regard to stagnant waters, they may be *depurators* where they are thus employed, and contribute to preserve a healthy action. It is true, as far as vegetables are concerned, especially grain, they appear to destroy, where they take up their residence, but when we discover the same or similar species, in sour paste or vinegar, they seem destined to consume substances that cease to be wholesome; and in fact, in all fluids, in which they usually so abound, they may be destined to fulfil a similar office, and it is a remarkable circumstance in their history confirmatory of this idea: that these animals, though animation in them is often suspended for a long time; when they swarm in infusions, having fulfilled their office, perish in a few days." 159.

But the principal *utility* of these countless armies of animalcules, after the enjoyment of their existence, is, doubtless, the supply of food for innumerable animals of a higher order than themselves. Thus it is well known that gold and silver fish require only a fresh supply of water every second or third day—their nutriment must therefore be drawn from the water, or rather from the animalculæ in the water. In this last may often be seen minute branchipods swimming about, sometimes with a bundle of eggs appended on each side. These, no doubt, enter into the bill of fare of the little finny prisoners.

In respect to the POLYPES, the most imperfect of them, as the sponges and some of the alcyons, appear to consist of a gelatinous mass, without any organs of prehension. They draw in and throw out water from which they derive nourishment. Most of them, however, have a mouth, with arms or tentacles. These are not only organs of sense, but serve for prehension, motion, and probably for a kind of respiration. The polypes are invariably aquatic animals.

The propagation of the common polypes of stagnant water is curious. It is by germs and cuttings. The former issue gradually from the body of the parent, like the branches from a trunk. The bud that forms the origin of the young one, is a continuation of the old one's skin—and its stomach of her stomach. The food passes from one to the other. After a time they detach themselves from the parent stem, and take on independent existence. In the course of a month, a single polype will thus prove the parent of a million of children ! They are also so redolent of vitality, that *cuttings* will become complete animals, thus increasing the other mode of propagation very considerably.

But the most wonderful polypes are the *madrepores* of Linneus, which are capable of erecting rocky reefs, submarine mountains, and extensive chains of islands, that emerge from the ocean, become covered with vegetation, and ultimately populated by animals and man. Vincent Rosa, an Italian naturalist, has described this species of coral, in the following words.

" 'From every cell,' says he, 'issues a cylindrical animal, resembling an intestine, transversely wrinkled, about half an inch long and two lines in diameter, and of which the upper extremity or mouth is surrounded by about twenty-two very short tentacles. These animals, which are pendent, because this madrepore is always fixed under the projections of the rocks, and vibrates at the will of the waves, are always of a lively orange colour, they contract as soon as they are touched, and they die upon being taken out of the water.' Whoever examines a fragment of the polypary of any of the varieties of white coral, will find it to consist of innumerable radiating tubes, variously intercepted, all of which appear to issue from a common base ; these are the receptacles of the general body of the polype, while the connected individuals with their blossoms inhabit an infinity of cells opening externally, from which the tentacles issue to collect their food." 181.

For purposes unknown to man, the Almighty has ordered these minute creatures, scarcely deserving the name of animals, to separate or elaborate calcareous particles from the briny wave, with which they build up their structures of stupendous magnitude. Each cell is inhabited by an individual animal—not, however, insulated from the parent body, but forming a part of a many-headed, and many-mouthed monster, which, at every oral orifice, is collecting the means of still farther increasing the coral palace—thus going on, till it has formed a habitation, not for itself alone, but for whole colonies of vegetables, animals, and the lords of the creation also!

We shall not follow our author in his speculations on the wisdom and goodness of God, in forming the class of madrepores. Hitherto the coral reefs have done little more than prove the destruction of many a gallant ship, and her crew. We are not, therefore, in a state to draw any very accurate inferences as to the utility of the madrepore's operations. Many navigators imagine that a day will come when the navigation of the tropical seas will be most hazardous, if not impracticable, on account of the coral reefs. We shall conclude this section of the subject with the following quotation, illustrating the force of INSTINCT in the lowest class of animated beings.

"There are two circumstances in the above account of the proceedings of these animals, that more particularly demonstrate Divine interposition. One is the precaution to which they have recourse when they build a circular reef in the sea, that they leave an opening in this part for the entrance of the tide and its reflux, so that a constant renovation of the waters takes place, without which they could not proceed in their operations, for want of their necessary aliment.

The other is, not only that they erect their buildings in the form best calculated to resist the action of the ocean, but also erect break-waters to strengthen the weakest points, and those from which the greatest danger is to be apprehended.

It is clear that beings so little organized, with scarcely any sense of feeling, are not sufficient of themselves to take these precautions, they must be directed and impelled by some power acting upon them; which, foreseeing the want, provides for it." 189.

The sixth chapter is on the RADIARIES. These appear to be a grade higher in organization than the polypes. The tendency to radiation begins even in the mineral kingdom, as is seen in the actinolites, pyrites, &c. It is observable in the blossoms of plants—in the seed in the earth—in the punctum saliens of the egg—in the fœtus—nay, even in sound, heat, &c. The whole world presents radiating bodies in every direction—suns shedding their rays upon attendant satellites, and the Almighty radiating his benevolent care and protection upon all!

As these radiaries cannot be propagated by slips or cuttings, they rise a link above the zoophytes. They are either divided into rays, like the star-fish, or with radiated crusts, as in the sea-urchins, or with radiations in their substance, as in the sea-nettle, or jelly-fish. They have not a terminal mouth surrounded by food-collecting tentacles, but one placed underneath the body. They are never fixed. They are divided by Lamarck into two orders—the gelatines and the echinoderms. In walking along the sea-shore, we sometimes see an animal of the gelatine tribe left by the waves, not much larger than a nutmeg, and nearly as transparent as the purest crystal. If injured by the least touch, it immediately dissolves. These lucid gems of the ocean sail gaily along, by means of their ciliated tails, receiving no injury in their frail organization. They are eminently phosphoric. When numerous, and in warm and calm nights, they afford a most brilliant spectacle. From their rotatory motion, they seem like globes of fire rolling along the surface of the ocean. They doubtless absorb animalcules from the water; and they are destined, in turn, to afford food to vast numbers of fishes—including the monarch of the floods—the whale himself, who does not disdain to dine and sup on these gems of jelly—more luxuriously, perhaps, than the alderman on turtle or white-bait! The sea-nettle is said to have an apparatus for raising or depressing itself in the sea. Many of them cause a sensation of stinging when touched—a low degree of galvanism. Their mode of propagation is not ascertained; but their fertility must be inconceivable, otherwise the whole race would be annihilated, in consequence of their fragility, and inability of resistance. They may be said to be created for the whales, and the whales for them. The masticating organs of the briny monster are precisely calculated for living on the gelatines.

We can spare but little space for the second order—ECHINODERMS. To look at a star-fish, one would be puzzled to account for its *progressive* motion. But they are furnished with tubes or suckers, by which they can attach themselves to substances, seize their prey, and effect locomotion in the waters. Their structure is very complicated. The jaws alone consist of 25 pieces, worked by 35 muscles—all having one common movement—and indicating that the animal subsists on a kind of food that is difficult of mastication.

The third and last section are the *fistuledans*. The sea anemonies are the most curious—animals which fix themselves on rocks, but have the power of locomotion. From a common base they send forth a number of stalks, terminating apparently in many-petalled flowers of various hues—so that people who have descended in diving-bells, have been astonished to see the submerged rocks covered with beautiful blossoms, of different colours, vying with the parterres of the gayest gardens. They come nearer the polypes in some respects; but their internal organization is much more advanced. They have a separate alimentary canal, surrounded by muscles, and even nervous nodules or ganglions, and ovaries. On the slightest danger, the animal contracts itself into the appearance of a mass of flesh. When inclined to change their place, they glide upon their base, or detach themselves entirely from the rocks, and commit themselves to the guidance of the waves.

TUNICARIES.—These are discussed in the 7th chapter. They are characterized as animals, either gelatinous or leathery, covered by a double tunic—the outer one analogous to the shell of the molluscans, organized and provided with two apertures, one for respiration and nutrition—the other for defecation. The inner envelope is like a mantle. The body is oblong, divided interiorly into many cavities without a head. The alimentary tube is open at both ends, and there is a ganglion sending nerves to the mouth and anus. The animals are either fixed or floating—single or aggregate. The mouth of all is surrounded with tentacles—exhibiting traces of a nervous system—and some of them even of a circulation. Some tribes of these creatures, as the *salpes*, have the property of uniting together, into a kind of community, like those of the bee, beaver, &c. on land—and this union is not fortuitous and irregular—but from birth, and in an undeviating order. It is the only example of such instinctive communities in the ocean. The object of the Creator, in endowing these animals with an instinct so singular, can only be conjectured. They are of a very frail nature (the salpes)

—they are so transparent, for instance, that their internal organs, their contents, and their movements, can all be distinguished—hence, perhaps, they would not have been able, as separate individuals, to resist the commotion of the waters in which they live.

The highest tribe of these live insulated from one another, and approach near the *molluscæ*.

"The pyrosomes are the largest of the phosphoric animals, the *Atlantic* species being about five inches long, and the *Mediterranean* sometimes attaining to the length of fourteen. Their power of emitting light is so great that in the night they cause the sea to appear on fire. Nothing can exceed the dazzling light and brilliant colours that these floating bodies exhibit—colours varying in a way truly admirable, passing rapidly every instant, from a dazzling red to saffron, to orange, to green and azure, and thus reflecting every ray into which the prism divides the light, or which is exhibited by the heavenly bow." 227.

Some of the tunicaries, when seized by the hand of the fisherman or sailor, eject the water which they contain with such force into the face of the assailant, that, in the astonishment of the moment, he suffers the animal to escape.

MOLLUSCÆ.—Although the great majority of this class of animals are aquatic, yet a considerable number of them are terrestrial, as any one may see after a shower of rain in the country—the snails or slugs.

This term comprehends a great number of animal forms, and it is difficult to select any character that is common to all. Conchology, indeed, is a study of great extent in itself—and when the structure and functions of the animals are added to the study of the shells themselves, it increases it tenfold. The molluscans may be designated as—" animal soft, without articulations —mantle bilobed, enveloping more or less the animal. Gills varying. A heart and circulation. No medullary chord, with ganglions, but a few scattered ganglions, from which the nerves issue to various parts. Body commonly defended by a calcareous shell, to which it adheres by only one or two points." The general habits of a large family of these seem to be that of *boring* and *burrowing*—many of them piercing wood, and even rock —others burrowing in the sand to a great depth. They are thus led by instinct to form a convenient cell or habitation for seizing their food, the precise nature of which is not yet ascertained—probably animalcular. The *sheathed* polype builds a house for itself, of matter elaborated in its own stomach, while the borer pierces wood, rock, &c. Our author thinks that Providence has willed these tiny animals to be the slow but sure agents, that effect immense changes on the earth's surface. Thus, rocks that form the apparently impregnable barrier of the land against the ocean are honeycombed by the polype, and eventually give way before the everlasting surges that assault them. This may be the case; but we confess that Mr. Kirby sees much farther than we can into the secret designs of the Almighty, in giving existence to the molluscans. The reverend author is more satisfactory when he describes the ingenious manner by which these little animals effect the various purposes to which their instincts prompt them. The account of the *razor-shell*, *arkle*, and *timber-borer* is very curious, but we must refer our readers to the volume itself. His description of the *timber-borer*, or shipworm, clashes somewhat with the beneficent purposes for which the molluscans are said to be created. These minute animals commit the most dreadful ravages on ships and houses, often bringing the habitations of man to the ground, destroying the inmates in an instant, when they thought themselves in security—or sinking a gallant ship, which, a few minutes previously, appeared perfectly sea-worthy ! Fortunately they have an aversion to fresh water, otherwise the foundations of our bridges would soon be undermined.

"The law laid down to the ship-worm is to hasten the decay of timber, that is out of its place, and may be denominated an unsightly encroachment upon the ocean—this is the law they must obey, and they make no distinction, whether it is disowned by all, or an important and valuable part of man's property. Their individual *object,* as has been stated above, is their own benefit, and they neither know that they obey a law of God, or injure man, but the Almighty by an irresistible agency impels them to it, and they fulfil the purposes of his Providence, at the same time that they provide for their own welfare." 245.

This is the proper statement of the case; the laws of instinct, like the laws which govern the whole solar system, were imposed never to be altered, whatever temporary or local inconvenience might occur. Yet it is believed by perhaps the great majority of the human race, and even in this enlightened country, that the Creator is perpetually interfering with his own laws, and dealing out special interpositions and dispensations, at the intreaty of individuals, or bodies of individuals—or, perhaps, from mere caprice ! Such doctrines are degrading alike to God and man !

But to return. The author adduces some curious particulars in the natural history of the oyster. Of all shell-fish, this is the most rude and unsightly—yet, in every age, it has been the one most in request. Like all other molluscans, it is hermaphrodite, and a single individual contains upwards of a million of eggs—so that one oyster may produce *twelve thousand barrels* of the same animal ! ! ! It is not likely, therefore, that the epicurean appetite of man shall ever annihilate the oyster ! They have other enemies besides man. It is said that certain species of crabs get into their calcareous citadels, by first introducing a piece of stone between the shells, to hinder their shutting. It is certain that the oyster defends itself against intrusive enemies, by squirting forcibly upon them water, kept in reserve within their shells. They keep out those which endeavour to pierce their tabernacles, by thickening the shell at the part attacked ! This, in itself, is a wonderful specimen of animal instinct !

The pearl oyster is interesting, from the ornament which it produces to gratify human vanity, and the early notice which it has obtained, both in sacred and profane history. But we dare not dwell on the almost infinite variety of the conchifers and molluscans—the pteropods, gastropods, trachepods, heteropods, &c.

"Enough has been said to prove that they have in no respect been neglected or overlooked by the Almighty Being who willed their existence, and who is ever watchful over the creatures of his hand, to provide them with all things necessary for their being, consistently with the ends he created them to serve." 266.

Cephalopods.—In the 10th chapter, our reverend and learned author arrives at a class of animated beings, remarkable, not only for their organization and habits, but for their position in the animal kingdom—as they seem to include elements from both the great divisions of that kingdom. From the VERTEBRATES, they take the beak, the eye, the tongue, the ear, the crop, the gizzard, an analogue of the spine, &c.—while, from their own sub-kingdom, they retain many of their remaining organs.

"From these circumstances it seems evident that the Creator has placed this tribe in a station which leads to very different and distant points in the animal kingdom, and that there is scarcely any but what may recognize in it one or more of its own peculiar features—yet at the same time it exhibits many characters, both in its most extraordinary outward form and in its internal organization, that are quite peculiar and *sui generis*, of which no animal at present known exhibits the slightest traces. To mention only its muscular apparatus, adapted to its unparalleled form; its system of circulation, carried on in the first Order by three distinct organs instead of one heart; and the wonderful complication of their tentacles, of the nerves that move them, and the vascular system that animates them." 305.

Cuvier denominated these wonderful animals CEPHALOPODS, from their feet being attached to their heads. Mr. Owen has divided this class into two orders, from the composition of their respiratory organs—namely, into those that have two branchiæ, and those that have four. The first includes those that have no shell—the second those that have one shell. In the first order is the cuttle-fish—one of the most wonderful of God's works.

"Its mouth is surrounded by eight long fleshy arms, or rather legs, somewhat conical in shape, and acute at the end, moved by innumerable nerves, furnished from numerous ganglions: these legs can bend in every direction with the utmost vigour and activity, their surface is furnished with many suckers, by which they can fix themselves strongly to any thing they wish to lay hold of, and by means of which, like the star-fish, they can move from place to place. When this animal walks, in this resembling also the star-fish and sea-urchin, it moves with its head and mouth downwards and its body elevated. It swims also and seizes its prey by means of these organs: besides these arms or legs, for they perform the functions of both, there is a pair of long organs, one on each side, having their origin between the first and second pair of legs, which are incrassated at the end, where, also, they are furnished with many suckers. Cuvier supposes they use these as anchors to maintain them in their station during tempests, and as prehensile instruments, by which they can seize their prey at a distance. In the centre of the legs is the mouth, surrounded by a tubular membranous lip, including a beak, consisting of two mandibles, like that of a paroquet; these mandibles or jaws are crooked, and the upper one fits into the lower as a sliding lid into a box. With these redoubtable jaws the cuttle-fish devours fishes, crustaceans, and even shell-fish, which receive a further trituration in its muscular crop and its gizzard. By means of the suckers on their legs and arms, they lay such fast hold of their prey as to deprive them of all power of motion; thus they master individuals much larger than themselves. The hard and often spinose crust of crabs or lobsters cannot withstand the action of their tranchant jaws, and they do not fear the gripe of their claws. Their large eyes, which resemble those of vertebrated animals, by their look of ferocity, are enough to create an alarm in the animals they pursue, and are said to see in the night as well as the day. So that although they are not like Pontoppidan's Kraken—the notion of which is thought to have been taken from a large cuttle-fish—half a league in circumference, so as to be mistaken for floating islands, yet they are really as tremendous animals, their size considered, as any that Providence has commissioned to keep within due limits the populace of the waters." 308.

They have three hearts. The principal one, seated in the middle, sends the blood through the arteries. The blood returns by a vein, which, dividing into two branches, carries it to the two lateral hearts, each of which sends it to the branchiæ for oxygenation, whence it returns to the middle heart. This is, as nearly as possible, the human circulation. These animals are remarkable for the possession of an organ which secretes a black fluid, with which they can render the water so turbid, on any appearance of danger, that they can thus conceal themselves from the enemy, or from the prey which they have in view.

We must pass over the pearly nautilus, which has excited so much attention from the time of Pliny to the present moment. By a single expansive organ, bearing resemblance to the foot of the snail, this animal is enabled to progress through the waters. Our author believes with Pliny, and many since his time, especially Bosc, that the nautilus spreads its light wings or sails to catch the breeze, and uses a kind of oar, at the same time, to accelerate its motions. Mr. Kirby next inquires into the object of Him who does nothing in vain, though the end may not always be evident, in producing these miniature monsters of the deep, so wonderfully organized, that they are unlike every other tribe of animals, and yet contain in them, as it were, the elements of those above and those below them.

"It appears, from the united testimony of every writer that has noticed them, that they have it in charge to keep within due limits, a tribe of animals, almost equally destructive with themselves, and which are armed also with weapons of offence, apparently equally terrific to their prey. It will be readily perceived that I am speaking of the *Crustaceans*, and of the formidable pincers with which they seize their prey. It must be a curious spectacle to see one of the larger poulpes attack a lobster; at first sight, we should think the latter most likely to master his assailant, covered as he is with a hard crust, and using adroitly his powerful forceps, we should feel sure that the cuttle-fish, with his soft body and oral organs equally soft, stood no chance against such an antagonist. But He who gave him his commission, has fitted him for the execution of it, his soft tentacular organs will bend in every direction, and the numerous suckers wherewith they are planted, by pumping out the medium that forms the atmosphere of marine animals, produce such a pressure wherever they are fixed, that, struggle as it may, it cannot disengage itself from the grasp of its assailant; and, by their flexibility, these organs can imitate the fishermen, and tie together the two pieces of the forceps, so that it cannot bite; thus, at last, it is brought within the action of the powerful beak of the cuttle-fish, which soon makes its way through its crust, and devours it shell and all. Even when at a distance, by means of its long arms, the cuttle-fish can lay hold of it and drag it towards it; and the poulpe, which has not these arms, makes up for it by having longer legs." 315.

One great object seems to be attained by the creation of this immense variety of molluscan animals—the production of CALCAREOUS MATTER. Even the shells of terrestrial testaceans must add, in no trifling degree, to the quantity of that matter on the surface of the earth, and probably make up for the continual waste or employment of it, so as to maintain the necessary equilibrium. But, in the ocean, the quantity, added to that produced by the corallines, must be prodigious! As the corallines raise reefs and islands, so the molluscans are raising the general bed of the ocean by their exuviæ!

The eleventh and twelfth chapters, on worms and annelidans, we must pass over, as not particularly interesting or novel; yet we cannot help offering one short extract respecting the *utility* (for our author is an *utilitarian* of the first water) of the *cerebral hydatid* of the sheep, and the *intestinal worms* of man.

"Though at first view the animals of which I have in the present chapter given some account seem to be altogether punitive, and intended as scourges of sinful man both in his own person and in his property, and their great object is hastening the execution of the sublapsarian sentence of death, yet this evil is not unmixed with good. Though fearful and hurtful to individuals, yet it promotes the general welfare by helping to reduce within due limits the numbers of man and beast. Besides, with regard to the Lord of the Creation, these things are trials that exercise his patience and other virtues, or tend to produce his reformation, and finally to secure to him an entrance into an immutable and eternal state of felicity, when that of probation is at an end, so that the gates of Death may be to him the gates of PEACE and REST." 331.

This reminds us of a presbyterian sermon which we once heard in Inverness, respecting cholera. The preacher assured his congregation, that the just man need not fear the epidemic which had then broken out, since he that worshipped the *true God* would escape DEATH. He concluded, however, with a sophism, that if a just man did happen to be carried off by cholera, it would not be DEATH, but a transition to eternal LIFE!

According to Mr. Kirby's doctrines, the medical art is exercised against the special workings of the Almighty! For surely if intestinal worms are "*intended* as scourges of sinful man," and their object is to hasten "the execution of the *sublapsarian* sentence of death," the calomel and scammony of the doctor must be an abomination in the sight of Heaven—or rather it must be perfectly useless, since it is not likely that the drugs of man will be able to arrest the direct decrees of his Creator! One more extract, and we shall close the volume. It contains matter of a very serious kind, and on which we shall venture a few comments.

"But to speak soberly, all we can safely affirm is, that He who decreed the *end* decrees the *means*, and these probably are physical ones under his direction. He it is who *guides the punitive animals* that he employs to their several stations. Is there not an omnipresent Deity, whose action is incessant, and co-extensive with his presence? He it is that, as the Prophet speaks, causeth it to *rain upon one city and not to rain upon another city*; that employs his instruments, both of benediction and punishment, according to his will. It is He, who by secret paths, and by means that mock our researches, conducts to their assigned station the animals in question. Every power of nature, every physical agent, is at His disposal. His is the earthquake and the volcano; *the lightning of the thunder*; the fire-damp of the mine; the overwhelming violence of the water flood; *the windy storm and tempest*: His is the wide-wasting *sword*, that destroys myriads, and *the pestilence that walketh in darkness*, and carries off millions; and He gives his commission to all his scourges *against individuals as well as against nations*, which they unconsciously execute and cannot exceed, for He saith to them, as to the raging sea, Hitherto shall ye come and no further, and here shall the work of destruction cease." 362.

This doctrine embraced, the inevitable inference is, that there is no such thing as an *evil principle* in nature. If the Almighty guides the sword, and gives "his commission to *all* his scourges—against individuals as well as against nations," that BEING of whom we have heard so much, and whom we hate so heartily, has been most slanderously abused and belied!

The office of SATAN is a complete sinecure, and, in these reforming days, will doubtless be abolished! What, then, has become of the evil passions and propensities of our nature? Where is the free-will of man, to embrace good or evil? Why are we said to transgress and break God's laws, when "the commission to *all* his scourges" comes from God himself? This doctrine is far worse than that of blind chance and fatalism! According to Mr. Kirby, it was the hand of the Almighty that fired the 25 muskets against Louis Philippe's cortège, and slaughtered the innocent and guilty indiscriminately! Where can be the justice of future rewards and punishments, if the sword, the dagger, the fire-damp, and the poison were guided by almighty, and, consequently, irresistible power? No, no.—The Almighty laid down general laws at the beginning, for man, for animals, for the elements, and even the Universe around them. These laws, being founded in infinite wisdom, require neither revision nor supervision. They are eternal and immutable. The animal creation he has bound by instinctive laws, from which they cannot deviate. To man he has given REASON, and, consequently, liberty to transgress some of his laws, both moral and physical, with the certainty of punishment for the transgression—thus making him a responsible BEING. But the doctrine of our author would take away all moral responsibility, since no will is left to the individual. There can scarcely be a more terrible or savage idea of an Omnipotent Power formed, than that which represents him as impelling irresistibly his creatures to deeds of good and evil, and then rewarding the one without merit, and the other without culpability! There is something frightful, too, in pointing out every calamity or affliction that befals an individual as a direct dispensation of the Almighty! and thus, in fact—

To deal damnation round the land,
On each *we judge* his foes!

But enough of theology. We have now run through the first volume, culling the most precious specimens of the work, and exhibiting half of the links that form the immense chain, reaching from the animalculæ in the drop of water, up to the half-reasoning elephant—nay, to the lord of the creation himself! In these times, when knowledge of all kinds is being diffused, with rapidity, through every ramification of society, we shall not deem it impolitic or useless to diverge a little, occasionally, into the collateral sciences, and thus diversify the severer studies of practical medicine. In our next number, we hope to complete the chain of communication between the lowest and highest grades of animated nature, and, in so doing, anticipate the approbation of our subscribers.

